# Community preparedness for earthquake disasters: A preliminary assessment of awareness and disaster infrastructure response in Cianjur, West Java-Indonesia

**DOI:** 10.12688/f1000research.143577.1

**Published:** 2024-04-08

**Authors:** Dewayany Sutrisno, Yatin Suwarno, Ati Rahadiati, Muhammad Iqbal Habibie, Prabu Kresna Putra, Hari Prayogi, Amien Widodo, Fathia Zulfati Sabrina, Ahmad Kosasih

**Affiliations:** 1 National Research and Innovation Agency, Cibinong, Cibinong, 16911, Indonesia; 2 Department of Geophysics, Faculty of Civil Planning and Geo Engineering, Institut Teknologi Sepuluh November, Surabaya, East Java, 60111, Indonesia; 3 Directorate of Disaster Risk Mapping and Evaluation, National Disaster Management Agency (BNPB), East Jakarta, Special Capital Region of Jakarta, 13120, Indonesia; 4 Center for Management and Geospatial Information Dissemination, Geospatial Information Agency, Cibinong, West Java, 16911, Indonesia

**Keywords:** Earthquake, community awareness, respondent’s survey, earthquake-resistant houses, Cianjur, Indonesia, disaster response, infrastructure

## Abstract

**Background:**

The danger of earthquakes poses a serious threat to people worldwide. One of the most significant challenges is preparing communities to cope effectively with this disaster. Therefore, understanding earthquake hazards is critically important for preparedness, mitigation, and an effective response to this threat. This report aims to observe and conduct a preliminary assessment of the degree to which community preparedness for earthquake disasters has been implemented.

**Methods:**

Empirical data were obtained from survey respondents and interviews. The respondents were members of a community affected by the Cianjur earthquake, which occurred on November 21, 2022. The data were analysed using the mean range approach, based on Likert scales. Additionally, the Spearman correlation method was employed to indicate the relationship between community awareness and infrastructure readiness.

**Result:**

Based on empirical evidence and preliminary analysis, it is evident that the preparedness of the community to respond effectively to earthquake catastrophes is inadequate. An apparent lack of readiness is observed in the inadequate construction of housing that fails to meet disaster standards, and the absence of disaster response facilities is notable.

**Conclusions:**

Based on our initial assumption, it appears that knowledge related to disaster resilience in this area has not been adequately disseminated or socialized. However, this premise requires further investigation.

## Introduction

The implementation of earthquake disaster risk reduction activities involving communities presents formidable challenges in numerous countries ( Hosseini *et al.*, 2014). Hence, implementing disaster preparedness measures is imperative for populations living in locations prone to earthquake hazards. However, ensuring earthquake safety cannot be guaranteed unless every member of the community possesses an in-depth awareness of the ramifications of earthquakes and has the knowledge to handle such situations ( Siawsh *et al.*, 2023). Indonesia, renowned for its high incidence of seismic activity worldwide scale ( Parwanto and Oyama, 2014), bears significant responsibility in the realm of earthquake prevention and mitigation. Consequently, evaluating community knowledge pertaining to earthquake hazards is a pivotal measure in the establishment of efficient policies for disaster preparedness and mitigation.

**Figure 1.**
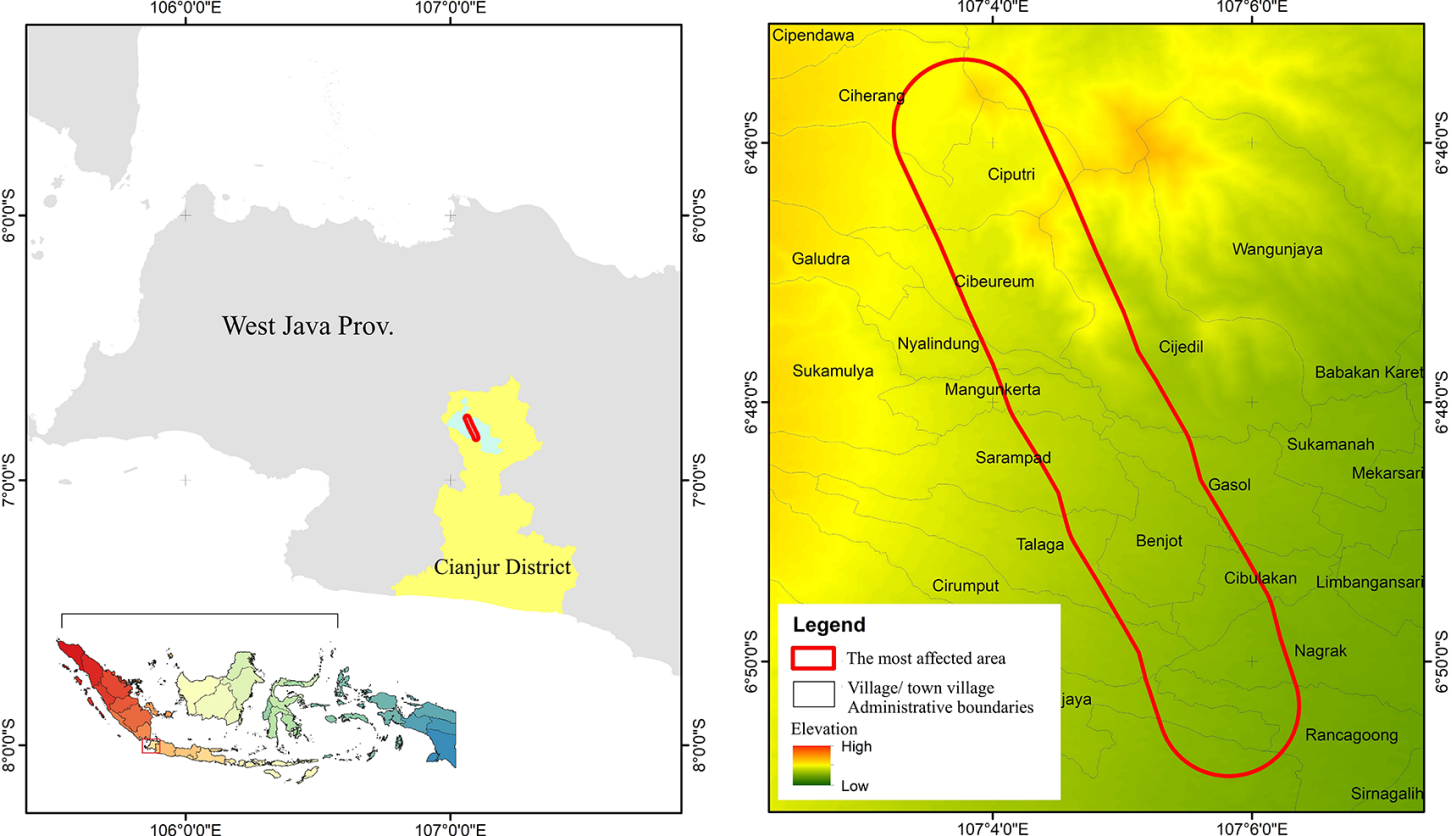
Location Cianjur’s November 2022 earthquake zones. Note: The red outlined area represents the most affected area, indicates the survey location

This report aims to observe and conduct a preliminary assessment of the degree to which community preparedness for earthquake disasters has been implemented. The report focuses on the community’s awareness of earthquake disasters and their relationship with the preparedness of the environment in which they reside, including housing and disaster response facilities. The earthquake discussed in this report was the Cianjur earthquake that occurred in West Java, Indonesia ( Figure 1). This seismic event took place on November 21st, 2022, with a magnitude of 5.6 ( Supendi *et al.*, 2023). The seismic event was characterized as a shallow or near-surface earthquake, and its destructive potential was not necessarily contingent on its magnitude. Instead, the seismic event not only resulted in the destruction of various structures, residences, and public facilities but also tragically led to the loss of at least 334 lives, injuries to 583 individuals, and the sudden displacement of 114,683 individuals who found themselves in a state of destitution ( BNPB, 2022; Antara news, 2022).

After a period of ten months following the occurrence, it is evident that the resolution of social issues and infrastructure difficulties remains incomplete. Many individuals continue to reside in temporary shelters including emergency tents and improvised educational facilities. The government has implemented housing initiatives to accommodate those living in regions affected by earthquakes, which are characterized by significant devastation. The extent of the success of this initiative will also be a part of this observation, as it is a crucial component of infrastructure disaster awareness.

## Methods

In this report, the hypothesis to be addressed is the level of disaster awareness and its influence on disaster preparedness infrastructure. Therefore, community awareness was investigated through respondent surveys and interviews.

The survey was conducted by disseminating questionnaires to residents in earthquake-affected areas, while interviews were exclusively conducted with officials from the four most severely impacted sub-districts, namely Rancagoong, Nagrak, Cibeureum, and Ciputri ( Figure 1). These sub-districts were identified as the most severely impacted by the Meteorology, Climatology, and Geophysical Agency (BMKG) in 2022. Both surveys and interviews were using the exact same sets of questions, and the results are processed in the same database and repository. The quantification of survey and interview participants is outlined in the subsequent subsection.

The steps of its implementation are as follows:

### Determining the eligibility of the respondents

The eligibility criteria for respondents include individuals residing in areas directly impacted by earthquake disasters, aged at least 20 years, proficient in the Indonesian language, from diverse professional backgrounds spanning from farmers to office workers, possessing a minimum educational level of high school, and willing and capable of participating as respondents in this research project.

### Determining the number of respondents

We employed a normal distribution algorithm to calculate the appropriate number of respondents, based on their respective proportions within the total population. We used 95% confidence level and a margin of error of 0.10. The algorithm is described as follows.

$$n=\frac{z^{2}.p\left(1-p\right)}{E^{2}}$$ (1)

where, *n* is the sample size, *z* is the confidence level, *p* is the population, and *E* is the error margin.

With the numbers of population being 500, the minimum number of respondents based on this calculation was 97.

### Ethical considerations

Before conducting the survey, ethical considerations regarding the participants were carried out. All participants were fully briefed about the purpose and nature of the research, including the assurance of confidentiality. Prior to conducting the surveys, informed consent was obtained from both the respondents and interview participants. This was accomplished by providing them with the option to either check a box or sign their assent on the first page of the questionnaire, which was utilized for both surveys and interviews.

For ethics approval, our institution requires that all decisions be formally sanctioned. The study has obtained ethical clearance approval under the reference Number 353/KE.01/SK/06/2023, issued on June 5, 2023, by the National Research and Innovation Agency of the Republic of Indonesia. Indeed, the authors have agreed to questionnaire’s content.

### Collecting the data

The survey and interview were based on three categories: individual’s awareness, building construction awareness, and disaster facilities and infrastructure. These categories were meticulously outlined in a questionnaire that employed uniform content and structure across both survey respondents and interview participants. The data collection was conducted from July 11th to July 13th, 2023, in the most earthquake-affected area of Cianjur Regency (see Figure 1). The questionnaire was disseminated to the community by village officials within the earthquake-affected areas, and the gathered responses were then returned to us by the village officials. Concurrently, the process of data collection for interviews was conducted face-to-face with the officials of the four affected sub-districts, *i.e.*, Rancagoong, Nagrak, Cibeureum, and Ciputri. The questions are:

*Individual’s awareness*

• Knowing you live in a seismically active location
• Understanding earthquake risk, including residence collapse and landslides
• Understanding the safely evacuate during an earthquake
• Having an earthquake emergency plan for the family
• Conversant with earthquake mitigation measures such as increasing awareness of potential household hazards, improving home conditions and emergency communication
• Have attended earthquake disaster awareness training or programs
• Monitoring earthquake information and warnings from credible sources

*Building criteria awareness*

• The destruction of residential structures resulting from seismic activity.
• Type of houses
• House typology in seismic context
• Acknowledge earthquake-resistant housing criteria
• Willingness to build earthquake resistant houses
• Knowledge of earthquake-resistant housing design and reconstruction utilizing local material
• Willingness to relocate if the previous location cannot be rebuilt.

*Disaster’s facilities and infrastructure*

• Muster points
• Evacuation zone
• Shelter
• healthcare facilities
• emergency response center
• Disaster information facilities.
• Communities self-manage disaster facilities and infrastructure.

### Analysis of the data

For the data analysis process, we used Microsoft Excel software from Office 2020. We utilized the following weighting scoring method:

$${\mathrm{ws}}_{i}=w_{i}(s_{i_{1}}+s_{i_{2}}+\cdots s_{i_{n}})$$ (2)

where *ws*_*i*_ is the total weighted score of variable *i*, $w_{i}$ the weights assigned to values of variable *i* and $s_{i_{1}}$ to $s_{i_{n}}$ are the score’s values of classes of variable *i* within the dataset.

As a new development, we transformed the weighted score value onto a Likert-4 scale utilizing the following formula:

$$s_{i}=\left(\left({\mathrm{wx}}_{i}-{\mathrm{wx}}_{r}\right)*\left(\frac{l-1}{{\mathrm{sc}}_{t}-{\mathrm{sc}}_{r}}\right)\right)+1$$ (3)

where $s_{i}$ is the value of the transformed ${\mathrm{wx}}_{i}$ , ${\mathrm{wx}}_{r}$ is the lowest value of ${\mathrm{wx}}_{i}$ , ${\mathrm{sc}}_{r}$ is the lowest score calculated from the low Likert scale (1) to the total number of n , l is the Likert scale (4), ${\mathrm{sc}}_{t}$ is the highest score calculated from the highest Likert scale to the total number of *n.*

We then modified the concept of Dacanay *et al.* (2018) to determine the community’s level of earthquake awareness and evaluate risk reduction infrastructure awareness ( Table 1).

**Table 1.** Level of awareness (modified from Dacanay *et al.*, 2018).

Scale	Median range	Categories	
4	3.51–4.00	Highly aware	All available
3	2.51–3.50	Aware	Partly available
2	1.51–2.50	Slightly aware	A bit available
1	1.00 –1.50	Not aware	Not available

### Correlation testing

To assess the correlation between disaster awareness and disaster preparedness infrastructure (houses and facilities), we used the Spearman’s correlation algorithm ( Spearman, 1904), there is:

$$r_{\mathrm{xy}}=\frac{\sum x^{2}+\sum y^{2}-\sum d^{2}}{2\sqrt{\sum x^{2}}\sum y^{2}}$$ (4)

where *x* represents the disaster awareness level, *y* represents infrastructure, *Σd* ( BNPB, 2022) is the sum of the squared differences between the ranks of the corresponding pairs of data points. In the event of duplicate data, the computations for variables *x* and *y* can be determined as follows.

$$\sum x^{2}=\frac{n\left(n^{2}\right)-1}{12}-\frac{t\left(t^{2}-1\right)}{12}$$ (5)

$$\sum y^{2}=\frac{n\left(n^{2}\right)-1}{12}-\frac{t\left(t^{2}-1\right)}{12}$$ (6)

Variable *n* represents the total number of data points, and *t* denotes the count of duplicate numbers.

A correlation coefficient > $r_{\text{table}0.05}\;$ indicates a positive relationship between the variables. A significance level of 0.05 is employed as a statistical tool to ascertain the statistical significance of the Spearman correlation coefficient ( *r*) between two variables within a dataset ( Zar, 1972).

The questionnaire was also subjected to validation and reliability testing using Cronbach’s alpha. If the calculated value of *r* was greater than 0.7, it was considered valid. The majority of validity values were > 0.7, indicating that it is valid, while the reliability value was 0.942, indicating that it is reliable ( Sutrisno, 2023).

## Results

The field survey data and its corresponding weighting can be seen on Open Science Framework ( Sutrisno, 2023).

The results of the data revealed that the majority of the community possessed a limited understanding of earthquake disasters and their impacts ( Table 2). This is indicated by issues related to knowing that they live in a seismically active location, understanding earthquake risk, knowing how to safely evacuate during an earthquake, monitoring earthquake information and warnings from credible sources, having an earthquake emergency plan for their family, being conversant with earthquake mitigation measures, and attending earthquake disaster awareness training.

**Table 2.** Respondents’ level of awareness on earthquake.

Issues of community awareness	Mean value	Categories
**Individual’s awareness**		
Knowing you live in a seismically active location	2.01	Slightly aware
Understanding earthquake risk, including residence collapse and landslides	1.76	Slightly aware
Understanding the safely evacuate during an earthquake	1.64	Slightly aware
Having an earthquake emergency plan for the family	1.14	Not aware
Conversant with earthquake mitigation measures such as increasing awareness of potential household hazards, improving home conditions and emergency communication	1.39	Not aware
Have attended earthquake disaster awareness training or programs	1.00	Not aware
Monitoring earthquake information and warnings from credible sources	1.68	Slightly aware
**Building criteria awareness**		
The destruction of residential structures resulting from seismic activity	2.36	Slightly aware
Type of houses	1.81	Slightly aware
House typology in seismic context	1.82	Slightly aware
Acknowledge earthquake-resistant housing criteria	1.68	Slightly aware
Willingness to build earthquake resistant houses	3.23	Aware
Knowledge of earthquake-resistant housing design and reconstruction utilizing local material	1.67	Slightly aware
Willingness to relocate if the previous location cannot be rebuilt	1.75	Slightly aware
**Disaster’s facilities and infrastructure**		
Muster points	1.91	A bit available
Evacuation zone	1.74	A bit available
Shelter	2.14	A bit available
Healthcare facilities	2.91	Partly available
Emergency response center	1.74	A bit available
Disaster information facilities	1.78	A bit available
Communities self-manage disaster facilities and infrastructure	1.42	Not available

We observed that a substantial number of participants exhibited a limited level of awareness regarding earthquake-resistant structures. Nevertheless, there exists a significant inclination towards constructing houses that can withstand earthquakes, which conflicts with the reluctance to relocate while inhabiting a region susceptible to earthquake hazards.

The Spearman analysis results demonstrated a correlation between the lack of awareness of earthquake disasters and the condition of settlements and readiness of infrastructure in their residential areas. The aforementioned calculations yield a Spearman coefficient of $r_{\mathrm{xy}}$ = 0.978 for the level of awareness pertaining to earthquake-resistant structures and a coefficient of $r_{\mathrm{xy}}$ = 0.962 for the level of preparation for disaster response facilities. The $r_{\text{table}0.05}$ for a confidence level of 0.05, Spearman’s rank is 0.714. In this case, the of $r_{\mathrm{xy}}$ > *r*_*table*0.05_; thus, it can be observed that there is a strong correlation between disaster awareness and earthquake-resistant houses and limitations in completing disaster resilience facilities.

**Figure 2.**
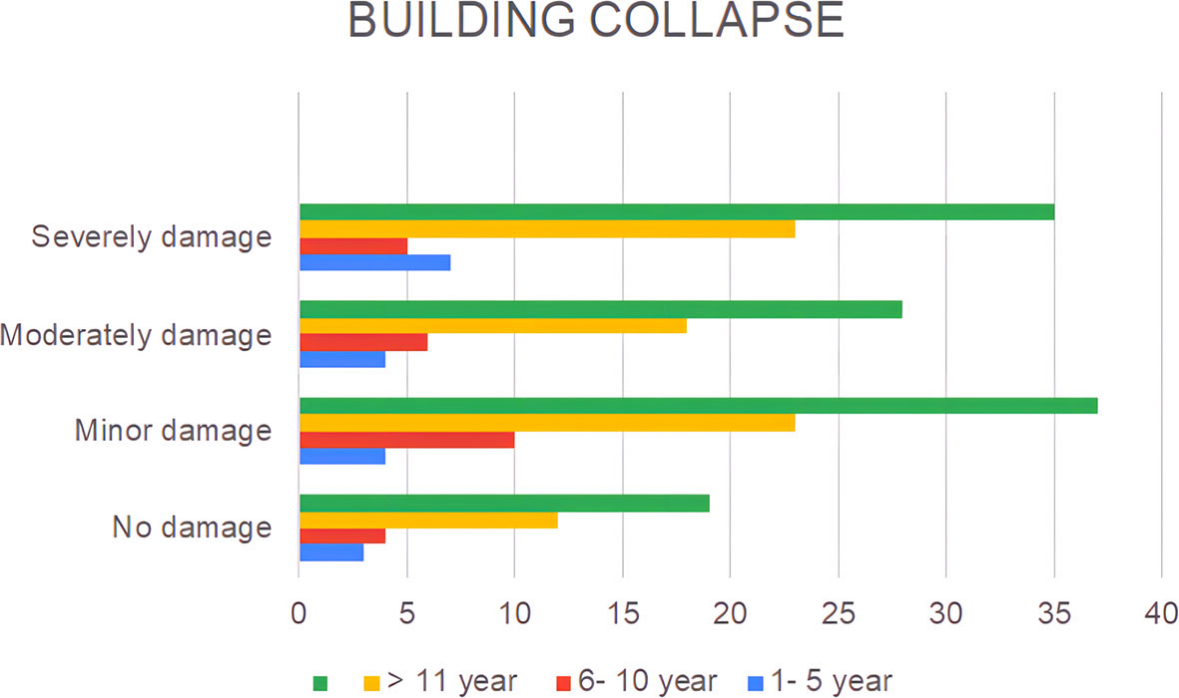
The extent of residential damage claimed by the respondents.

A lack of public awareness of earthquake disasters and the condition of the buildings in which they reside can also be observed at the level of building damage, as shown in Figure 2.

The results show that only 16% of the houses remained undamaged, while the rest experienced damage ranging from minor (31%), moderate (24%), to severe (29%) ( Figure 2). The observed phenomenon can be ascribed to the inherent heterogeneity in the construction quality of buildings with regard to their capacity to satisfy the earthquake-resistant criteria.

## Discussion

The results mentioned above indicate a positive association between the population’s inadequate knowledge of earthquake hazards and standards of earthquake-resistant housing and disaster response facilities. We also see two possibilities related to our previous findings: knowledge related to disaster resilience in this area has not been well disseminated or socialized, or they are unaware that they reside in earthquake-prone locations. This is evidenced by several issues such as the absence of sufficient planning for mitigating disasters, insufficient public education on appropriate measures to be performed during earthquakes, limitations in comprehending and equipping disaster resilience infrastructure, and a lack of understanding related to earthquake-resistant housing. Understanding earthquake infrastructure is crucial because the fatalities of earthquakes are commonly caused by building collapse ( Markušic *et al.*, 2020).

The period of 10 months following the catastrophe has proven to be a significant educational opportunity for all individuals involved. The government has initiated the process of providing guidance to the community to facilitate the reconstruction of their homes in alignment with the prescribed criteria ( World Bank, 2021; BSN, 2019). This was demonstrated by the level of public awareness of the construction of earthquake-resistant housing, particularly in light of the accessibility of financial assistance and the provision of guidance by the local housing authority. Regrettably, the provision of aid is not accompanied by educational initiatives pertaining to disaster preparedness or building infrastructure for disaster response.

A limitation of this study was the absence of in-depth interviews regarding sociocultural issues. Disaster-affected areas are characterized by features of agricultural and mountainous tourism. We assume that an agrarian society’s strong attachment to farming practices contributes to their reluctance to relocate far from agricultural land. The potential impact of tourism on the limited availability of traditional Sundanesse wooden houses in this area is also considered. These wooden houses were designed to withstand local natural phenomena and exhibit earthquake resistance ( Dutu, 2021). Another limitation is that socio-culture is a periodic public awareness campaign, the provision of training programs for disaster preparation, the incorporation of earthquake education into the school curriculum, and the active involvement of local populations in disaster simulations and drills. We believe that these aspects require further research.

## Data Availability

Open Science Framework: https://doi.org/10.17605/OSF.IO/UJ68Z ( Sutrisno, 2023). The project contains the following underlying data:
- Recapitulation of surveys_rw.csv (Raw data from questionnaire and interviews) - Matrix of Scored Analysis.csv (Analysed data) - Reability Test_analysis.csv (Analysed data) - Validity Test_analysis.csv (Analysed data) Open Science Framework: https://doi.org/10.17605/OSF.IO/UJ68Z ( Sutrisno, 2023). The project contains the following extended data:
- Quisitionnaire_CC awarness2023.pdf (Questionnaire) Data are available under the terms of the Creative Commons Attribution 4.0 International license (CC-BY 4.0).

## References

[ref1] Antara news: Gempa-cianjur-korban-meninggal-bertambah-menjadi-334-orang. 2022. Accessed 3 December 2022. Reference Source

[ref2] BNPB: [UPDATE]. 327 Orang Meninggal Dunia Pasca Gempa Cianjur. 2022. Accessed 7 July 2023. Reference Source

[ref14] BSN (Badan Standarisasi Nasional). Standar Nasional Indonesia (SNI 1726): *Tata cara perencanaan ketahanan gempa untuk struktur bangunan gedung dan nongedung.* Jakarta: BSN;2019.

[ref3] Dacanay JM Mauro MGB Sandoval JA : Community awareness on earthquake and assessment on earthquake risk reduction practices in a Philippine municipality. *Int. J. Appl. Sci.* 2018;1(2):77–83. 10.30560/ijas.v1n2p77

[ref4] Dutu: Chapter 14 - An engineering view of traditional timber frames with infills in Romania. Masonry Construction in Active Seismic Regions Woodhead Publishing Series in Civil and Structural Engineering. 2021;2021:277–420. 10.1016/B978-0-12-821087-1.00005-3

[ref5] Hosseini KAKA Hosseini M Izadkhah YO : Main challenges on community-based approaches in earthquake risk reduction: Case study of Tehran, Iran. *Int. J. Disaster Risk Reduct.* 2014;8:114–124. 10.1016/j.ijdrr.2014.03.001

[ref6] Markušic S Stanko D Korbar T : The Zagreb (Croatia) M5.5 earthquake on 22 March 2020. *Geosciences.* 2020;10(7):252. 10.3390/geosciences10070252

[ref7] Parwanto NB Oyama T : Statistical analysis and comparison of historical earthquakes and tsunami disasters in Japan and Indonesia. *Int. J. Disaster Risk Reduct.* 2014;7:122–141. 10.1016/j.ijdrr.2013.10.003

[ref8] Siawsh N Peszynski K Vo-Tran H : Toward the creation of disaster-resilient communities: The Machizukuri initiative – The 2011 Tōhoku Great East Japan Earthquake and Tsunami. *Int. J. Disaster Risk Reduct.* 2023;96:103961. 10.1016/j.ijdrr.2023.103961

[ref9] Supendi P Winder T Rawlinson N : A conjugate fault was revealed by the destructive Mw 5.6 (November 21, 2022) Cianjur earthquake, West Java, Indonesia. *J. Asian Earth Sci.* 2023;257:105830. 10.1016/j.jseaes.2023.105830

[ref10] Spearman C : Proof and measurement of the association between two things. *Am. J. Psychol.* 1904;15(1):72–101. 10.2307/1412159 3322052

[ref11] Sutrisno D : Community perception on disasters.[Dataset]. *Open Science Framework.* 2023. 10.17605/OSF.IO/UJ68Z

[ref12] World Bank: *Building Indonesia’s Resilience to Disaster: Experiences from Mainstreaming Disaster Risk Reduction in Indonesia Program. A report for the implementation of P122240 – Mainstreaming Disaster Risk Reduction in Indonesia Phase II Programmatic Advisory Services Analytics.* Jakarta: World Bank;2021. Reference Source

[ref13] Zar JH : Significance Testing of the Spearman Rank Correlation Coefficient. *J. Am. Stat. Assoc.* 1972;67(339):578–580. 10.1080/01621459.1972.10481251

