# Supplementary material for: Community preparedness for earthquake disasters: A preliminary assessment of awareness and disaster infrastructure response in Cianjur, West Java-Indonesia
Source: F1000Res. 2025 Feb 20;13:256. Originally published 2024 Apr 8. [Version 3] doi: 10.12688/f1000research.143577.3 (PMC11894365; doi:10.12688/f1000research.143577.3)
Supplement: Peer review history

# Reviewer report: Reviewer response for version 3

**DOI:** 10.5256/f1000research.176532.r367850

**Published:** 2025-03-10

**Authors:** Totok Doyo Pamungkas

**Affiliations:** 1 Universitas Pendidikan Indonesia, Bandung, West Java, Indonesia

No further comments

Is the work clearly and accurately presented and does it cite the current literature?

No

If applicable, is the statistical analysis and its interpretation appropriate?

I cannot comment. A qualified statistician is required.

Are all the source data underlying the results available to ensure full reproducibility?

Partly

Is the study design appropriate and is the work technically sound?

No

Are the conclusions drawn adequately supported by the results?

No

Are sufficient details of methods and analysis provided to allow replication by others?

Partly

Reviewer Expertise:

geography education, disaster preparedness, geology and geophysics, sustainable development

I confirm that I have read this submission and believe that I have an appropriate level of expertise to confirm that it is of an acceptable scientific standard.

# Reviewer report: Reviewer response for version 2

**DOI:** 10.5256/f1000research.169729.r345361

**Published:** 2024-12-26

**Authors:** Totok Doyo Pamungkas

**Affiliations:** 1 Universitas Pendidikan Indonesia, Bandung, West Java, Indonesia

1. This paper needs more or less ten references about community preparedness for earthquake disasters, especially in West Java, with the same characteristic

 2. Study design needs more clarity such as workflow

 3. Indicator of individual awareness measurement based on reference or by the researcher?, where do the following aspects:

 1) knowledge; 2) attitude; 3) behavior. has been add

 4. The characteristics of respondents must show and clearly explain about experience/ years of living in the area is one of the important indicator

 5. The conclusions drawn are not based on the final results but tend to argue and assume based on researchers

Is the work clearly and accurately presented and does it cite the current literature?

No

If applicable, is the statistical analysis and its interpretation appropriate?

I cannot comment. A qualified statistician is required.

Are all the source data underlying the results available to ensure full reproducibility?

Partly

Is the study design appropriate and is the work technically sound?

No

Are the conclusions drawn adequately supported by the results?

No

Are sufficient details of methods and analysis provided to allow replication by others?

Partly

Reviewer Expertise:

geography education, disaster preparedness, geology and geophysics, sustainable development

I confirm that I have read this submission and believe that I have an appropriate level of expertise to confirm that it is of an acceptable scientific standard, however I have significant reservations, as outlined above.

# Response

**Published:** 2024-12-31

**Authors:** Dewayany Sutrisno

**Affiliations:** Geospatial Research Center, Badan Riset dan Inovasi Nasional Republik Indonesia, Central Jakarta, Jakarta, Indonesia

**RESPOND TO THE REVIEWER:**

1. This paper needs more or less ten references about community preparedness for earthquake disasters, especially in West Java, with the same characteristic:

Thank you for your feedback. Ten references related to community preparedness for earthquake disasters, with the majority focusing on locations in West Java, have been incorporated into the chapter Instruments Used: Questionnaire and Discussion and supplemented with appropriate references

  

1. Study design needs more clarity such as workflow:

Thank you for your suggestion. The workflow study design has been included in the Methods chapter as Figure 2.

  

1.  Indicator of individual awareness measurement based on reference or by the researcher?, where do the following aspects:
  1) knowledge; 2) attitude; 3) behavior.

Thank you for the feedback. Indicators of individual awareness are based on measurable criteria. The aspects of 1) knowledge, 2) attitude, and 3) behavior have been incorporated into the three main sections: individual awareness, building construction awareness, and disaster facilities and infrastructure. These are elaborated as follows:

  

- Individual awareness relates to the knowledge a community possesses about earthquakes and their associated impacts, as well as the extent to which earthquake disaster mitigation efforts are understood by the population.
- Building construction awareness refers to residents' attitudes toward the construction of housing structures that can protect them from the impacts of earthquake disasters (Hariyanto et al., 2020; Putranesa et al., 2023).
- Disaster facilities and infrastructure pertain to the behavior of a community in responding to disasters, including their efforts in preparing earthquake-resilient infrastructure independently (Willar et al., 2022).

This has been included in the chapter Instruments: Questionnaire."

  

1. The characteristics of respondents must show and clearly explain about experience/ years of living in the area is one of the important indicator

Thank you for your suggestion. The author has enhanced the description of respondent characteristics in the Samples chapter by adding the following statement:

 The eligibility criteria for respondents include individuals residing in areas directly impacted by earthquake disasters, particularly those who are native residents with ancestral ties to the area, aged at least 20 years, proficient in the Indonesian language, from diverse professional backgrounds spanning from farmers to office workers, possessing a minimum educational level of high school for village officials and any level of education for common survey respondents, and willing and capable of participating as respondents in this research project

  

1. The conclusions drawn are not based on the final results but tend to argue and assume based on researchers: Thank you for your suggestion. A conclusion chapter has been added to the manuscript, which was previously absent. The initial omission of the conclusion chapter was due to our adherence to the existing report format guidelines.

- **Is the work clearly and accurately presented and does it cite the current literature?; **The author has incorporated recent literature, with 16 out of 24 references being articles published after 2020
- **Is the study design appropriate and is the work technically sound?** The workflow study design has been included in the Methods chapter as Figure 2.
- **Are sufficient details of methods and analysis provided to allow replication by others?**The method has been supplemented with a workflow and its corresponding explanation
- **If applicable, is the statistical analysis and its interpretation appropriate?**I cannot comment. A qualified statistician is required.
- **Are all the source data underlying the results available to ensure full reproducibility?**In accordance with the journal's guidelines, the data is stored in a repository with the link provided in the manuscript.
- **Are the conclusions drawn adequately supported by the results?** A conclusion chapter has been added to the manuscript, which was previously absent. The initial omission of the conclusion chapter was due to our adherence to the existing report format guidelines.

# Reviewer report: Reviewer response for version 2

**DOI:** 10.5256/f1000research.169729.r311003

**Published:** 2024-09-10

**Authors:** Ayse Nuray Karanci

**Affiliations:** 1 TOBB Ekonomi ve Teknoloji Üniversitesi, Ankara, Turkey

The revised version of this paper is acceptable the authors have answered my queries and have revised the paper accordingly. Therefore I approve this version to be indexed.

Is the work clearly and accurately presented and does it cite the current literature?

No

If applicable, is the statistical analysis and its interpretation appropriate?

Partly

Are all the source data underlying the results available to ensure full reproducibility?

Partly

Is the study design appropriate and is the work technically sound?

No

Are the conclusions drawn adequately supported by the results?

No

Are sufficient details of methods and analysis provided to allow replication by others?

No

Reviewer Expertise:

Disaster prepaparedness

I confirm that I have read this submission and believe that I have an appropriate level of expertise to confirm that it is of an acceptable scientific standard.

# Reviewer report: Reviewer response for version 1

**DOI:** 10.5256/f1000research.157253.r297088

**Published:** 2024-07-04

**Authors:** Ayse Nuray Karanci

**Affiliations:** 1 TOBB Ekonomi ve Teknoloji Üniversitesi, Ankara, Turkey

Although this is an interesting study of a population exposed to a recent earthquake there are important weaknesses in the manuscript. These are as follows:

 Introduction:

 Concept of awareness not only on earthquake hazards but also on adaptive behaviors needs to be discussed. There are numerous theoretical models linking awareness to adaptive behaviors. The introduction fails to discuss these theories.

 The terms used like disaster preparedness infrastructure needs to be specified.

 The hypothesis of the study needs to be more clearly given.

 Method: This section needs to be organized as sample (for the questionnaire and interviews) Instruments used and procedure.

 Sample : Number of participants, the sociodemographic characteristics of the survey and interview participants need to be given in a table (age gender education etc.)

 Participants: Minimum of high school education was chosen. The authors need to explain the reason behind this.

 Survey instrument: Information on how it was developed the response format for the questions and the time frame to which the questions address, and the psychometric properties like Cronbach alpha values needs to be given.

 Procedure: For data collection needs to be given. The authors have done some interviews but no information is given on the results and the procedure.

 Results:

 Due to a lack of specific hypothesis the results and the analysis does not seem clear. A multiple regression analysis for adaptive behavior predictors may make the results more comprehensible.

 Discussion: Needs to be extended after considering all the above points. As it stands it is not clear how the authors reached these conclusions and their recommendations .

Is the work clearly and accurately presented and does it cite the current literature?

No

If applicable, is the statistical analysis and its interpretation appropriate?

Partly

Are all the source data underlying the results available to ensure full reproducibility?

Partly

Is the study design appropriate and is the work technically sound?

No

Are the conclusions drawn adequately supported by the results?

No

Are sufficient details of methods and analysis provided to allow replication by others?

No

Reviewer Expertise:

Disaster prepaparedness

I confirm that I have read this submission and believe that I have an appropriate level of expertise to state that I do not consider it to be of an acceptable scientific standard, for reasons outlined above.

# Response

**Published:** 2024-07-23

**Authors:** Dewayany Sutrisno

**Affiliations:** Geospatial Research Center, Badan Riset dan Inovasi Nasional Republik Indonesia, Central Jakarta, Jakarta, Indonesia

**Author Response to THE REVIEWER -**

**Reviewer Comment:**

 --------------------------------

 Although this is an interesting study of a population exposed to a recent earthquake there are important weaknesses in the manuscript. These are as follows:

**Introduction:**

 Concept of awareness not only on earthquake hazards but also on adaptive behaviors needs to be discussed. There are numerous theoretical models linking awareness to adaptive behaviors. The introduction fails to discuss these theories.

 --------------------------------

**Author Response:** 

 Thank you for your feedback. Indeed, there are some sentences in this paper that may have led to misunderstandings. This paper is part of a study aimed at understanding community perceptions regarding earthquake-resistant houses promoted by the Indonesian government, by considering that casualties from earthquakes are generally caused by building collapses. It is indicated by the title, “Community preparedness for earthquake disasters: A preliminary assessment of awareness and disaster infrastructure response in Cianjur, West Java-Indonesia". We apologize, but in this paper, we focus more on raising community awareness about living in earthquake-prone areas, as well as the need for understanding earthquake-resistant houses and related infrastructure as part of community preparedness. Therefore, we did not conduct research related to community adaptability, as this would be more aligned with studies on community resilience.

      Regarding earthquake-resistant buildings, their ancestors had local wisdom by constructing traditional wooden houses in response to disaster-related concerns. However, the modernization of housing has altered the community's patterns, leading to limited knowledge about earthquake-resistant housing and other disaster preparedness facilities. This is a common issue in various earthquake-prone villages in Indonesia.

      Following your feedback, the revisions can be seen in the Introduction, paragraphs 1 and 3."

 “ …….Consequently, evaluating community knowledge pertaining to earthquake hazards is a pivotal measure in the establishment of efficient policies for disaster preparedness and mitigation, such as the awareness of living on the earthquake prone area, earthquake-resistant housing and its related infrastructures”

 and

 …..” The extent of the success of this initiative will also be a part of this observation, as it is a crucial component of infrastructure disaster awareness. Since most of the casualties in an earthquake have been determined to be deaths caused by buildings collapsing (Sutrisno et al, 2024)…”

**Reviewer Comment:**

 --------------------------------

 The terms used like disaster preparedness infrastructure needs to be specified.

 --------------------------------

**Author Response:** 

 Thank you for your feedback. The term 'disaster preparedness infrastructure' that we refer to is specified as

1. Earthquake-Resistant Buildings: Structures designed to withstand the shocks and damage caused by earthquakes.
2. Evacuation Facilities: Safe and organized locations for evacuating residents during disasters, including evacuation routes and temporary shelters
3. disaster facilities: such as health care facilities and emergency respond center.
4. Training and Education: Programs that train and educate the community on how to respond correctly and effectively to disasters

See the Sub chapter of instruments used and the questionnaire

 And

 the last paragraph of the introduction

 “….The disaster infrastructure referred to in this article includes buildings, particularly earthquake-resistant houses, as well as evacuation facilities such as evacuation roads, muster points, health facilities, and community training programs.”

**Reviewer Comment:**

 --------------------------------

 The hypothesis of the study needs to be more clearly given.

 --------------------------------

**Author Response:** 

 The hypothesis to be tested in this study is that the level of disaster awareness has a significant influence on disaster preparedness infrastructure. To clarify, the details of this hypothesis are as follows:

1. Level of Disaster Awareness: This refers to the extent to which individuals or communities understand and recognize the risks and hazards associated with earthquakes in their living environment.
2. Disaster Preparedness Infrastructure: This encompasses all facilities, systems, and equipment prepared to deal with disasters, including earthquake-resistant buildings, early warning systems, evacuation facilities, emergency supply storage, and community training programs.

This hypothesis posits that the higher the level of disaster awareness in a community, the better and more comprehensive the available disaster preparedness infrastructure.

**Reviewer Comment:**

 --------------------------------

**Method**:

 This section needs to be organized as sample (for the questionnaire and interviews) Instruments used and procedure.

 Sample : Number of participants, the sociodemographic characteristics of the survey and interview participants need to be given in a table (age gender education etc.)

 --------------------------------

**Author Response:** 

 Thank you for your feedback. We have restructured the methodology, as can be seen in the Methods chapter of this paper. In accordance with the reviewers’ suggestions, this section is divided into three subsections: samples, instruments used, and procedures.

 Regarding the data, the tables can be accessed by clicking the link provided in the Data Availability chapter of this paper (Repository Data).

**Reviewer Comment:**

 --------------------------------

 Participants: Minimum of high school education was chosen. The authors need to explain the reason behind this.

 --------------------------------

**Author Response:** 

 Thank you for your feedback. Some word is missing, which we have corrected in the samples sub-chapter.

 “……..The eligibility criteria for respondents include individuals residing in areas directly impacted by earthquake disasters, aged at least 20 years, proficient in the Indonesian language, from diverse professional backgrounds spanning from farmers to office workers, possessing a minimum educational level of high school for village’s officials and any level of educations for respondents, and willing and capable of participating as respondents in this research project….”

**Reviewer Comment:**

 --------------------------------

 Survey instrument: Information on how it was developed the response format for the questions and the time frame to which the questions address, and the psychometric properties like Cronbach alpha values needs to be given.

 --------------------------------

**Author Response:** 

 Thank you for your feedback. The methodology has been restructured according to the reviewers' suggestions.

 Development of Response Format:

 The response format for the questions was developed based on a literature review and discussions with experts in the field. We used a 4-point Likert scale to measure respondents' readiness for earthquake disasters. The questions in the questionnaire were designed to cover the respondents' experiences and knowledge from the period before the disaster up to ten months after the disaster. This was done to ensure that the data collected is relevant and current. To measure the internal consistency of the questionnaire, we calculated Cronbach's alpha values for each section of the questionnaire, aiming for values > 0.7 to establish validity. These values indicate that the questionnaire has good internal consistency and is reliable for measuring the intended research objectives.

**Reviewer Comment:**

 --------------------------------

 Procedure: For data collection needs to be given. The authors have done some interviews but no information is given on the results and the procedure.

 --------------------------------

**Author Response:** 

 Thank you for the feedback. The interviews used the same format as the respondent survey. We have made corrections in the data collection subsection

 “…..Concurrently, the process of data collection for interviews was conducted face-to-face with the officials of the four affected sub-districts, *i.e.*, Rancagoong, Nagrak, Cibeureum, and Ciputri. As previously explained in the Ethics Clearance chapter, the research background was first explained, followed by obtaining consent to participate, and then proceeding with the interview process…”

**Reviewer Comment:**

 --------------------------------

**Results:**

 Due to a lack of specific hypothesis the results and the analysis does not seem clear. A multiple regression analysis for adaptive behavior predictors may make the results more comprehensible.

 --------------------------------

**Author Response:** 

 Thank you for your feedback. We recognize that the lack of specific hypotheses can make the results and analysis appear unclear. As we mentioned earlier, this research focuses on community preparedness for earthquake disasters, specifically to assess awareness of living in the prone zone, the importance of earthquake-resistant houses and its supporting infrastructure. Therefore, we did not conduct an analysis of adaptive behaviour.

**Reviewer Comment:**

 --------------------------------

**Discussion:**

 Needs to be extended after considering all the above points. As it stands it is not clear how the authors reached these conclusions and their recommendations.

 --------------------------------

**Author Response:** 

 Thank you for your feedback. We have completed and revised it according to the objectives of this research.
